# Effects of Sodium Azide on the Abundance of Prokaryotes and Viruses in Marine Samples

**DOI:** 10.1371/journal.pone.0037597

**Published:** 2012-05-18

**Authors:** Christian Winter, Marie-Emmanuelle Kerros, Markus G. Weinbauer

**Affiliations:** 1 Department of Marine Biology, University of Vienna, Vienna, Austria; 2 Microbial Ecology and Biogeochemistry Group, Centre National de la Recherche Scientifique, Laboratoire d'Océanographie de Villefranche, Villefranche-sur-Mer, France; 3 Laboratoire d'Océanographie de Villefranche, Université Pierre et Marie Curie-Paris 6, Villefranche-sur-Mer, France; University of Illinois, United States of America

## Abstract

Flow cytometry is set to become the standard method for enumerating prokaryotes and viruses in marine samples. However, the samples need to be flash-frozen in liquid nitrogen directly after aldehyde fixation. Because liquid nitrogen may not always be available, we tested the potential of sodium azide as a preservative for prokaryotes and viruses in marine samples as a possible alternative. For that we conducted incubation experiments with untreated and sodium azide treated marine water samples at 4°C and room temperature. The data indicate that sodium azide cannot be used to maintain marine samples used for the enumeration of prokaryotes and viruses.

## Introduction

Given the ubiquitous distribution of prokaryotes and viruses in marine environments, measurements of their abundance have become important parameters for many marine studies. Since only a small fraction of prokaryotes found in the ocean can be cultured on conventional media, it is necessary to enumerate prokaryotes directly. Current methods involve staining of prokaryotes with fluorochromes, followed by determining their abundance using epifluorescence microscopy [1], [2] or flow cytometry [3]. Similarly, viral abundance can be determined directly using transmission electron microscopy [4] or, upon staining with fluorochromes, using epifluorescence microscopy [5], [6] and flow cytometry [7]. Common to all of these direct-counting techniques is the need for the samples to be preserved with aldehyde-based fixatives. It was demonstrated that storage conditions of aldehyde-fixed samples for the enumeration of prokaryotes [8], [9] and viruses [10] are critical and may substantially alter abundances. Especially, storage of fixed samples at 4°C leads to a substantial decrease in prokaryotic abundance within days and for viruses within hours.

Sodium azide (NaN_3_) is a potent bacteriostatic that is frequently used to protect a diverse array of stock solutions (e.g., antibodies) and samples (e.g., milk, fecal specimens) from prokaryotic contaminants. NaN_3_ binds to heme-iron (cytochrome oxidase, catalase; [11]) leading to chemical asphyxiation of affected cells. However, the bacteriostatic effects of NaN_3_ appear to be limited to Gram-negative *Bacteria*, whereas Gram-positive *Bacteria* are largely resistant to the compound [12], [13]. Based on studies conducted mostly with non-marine, archaeal isolates obtained from environments such as acidic hot springs or solar salterns, the effects of NaN_3_ on archaeal metabolism vary among taxa from susceptible to resistant [14].

Currently, samples for the concomitant enumeration of prokaryotes and viruses need to be processed immediately after aldehyde fixation in order to avoid decay of prokaryotes and viruses [8], [9], [10]. This involves either the preparation of filter slides and subsequent storage at −20°C for epifluorescence microscopy or flash-freezing of samples in liquid nitrogen and storage at −80°C for flow cytometry. However, this may not be possible in every case (e.g., large numbers of samples obtained at once, sample transport). Since flow cytometry is set to replace epifluorescence microscopy as the new standard method [15], availability of liquid nitrogen used to prepare flow cytometry samples for storage may become a limiting factor. The aim of this study was to test the potential of NaN_3_ as a preservative for marine samples to obtain accurate numbers of prokaryotes and viruses. However, our data indicate that preservation with NaN_3_ is not a viable alternative for aldehyde-fixation and flash-freezing.

## Results and Discussion

### Treatment effects

**Figure 1:**
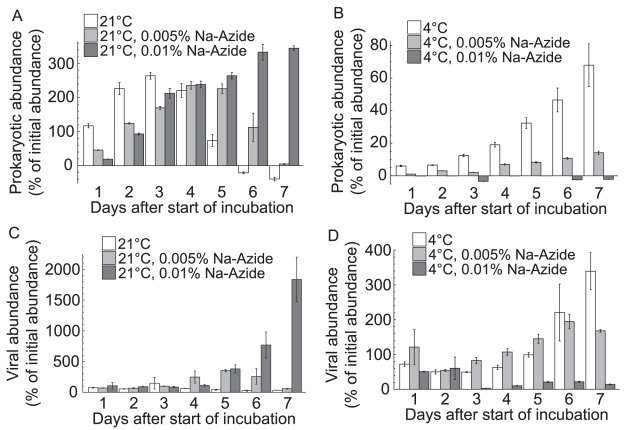
Example of the temporal development of prokaryotic and viral abundance. The figure shows the changes in prokaryotic and viral abundance relative to initial abundances in Exp. 1 (5 m depth, DYFAMED station) for each treatment. The data are given as the average of triplicate incubations and error bars represent standard deviations.

Initial prokaryotic and viral abundance was 7.7×10^5^ ml^−1^ and 6.3×10^6^ ml^−1^ for Exp. 1, 7.9×10^5^ ml^−1^ and 24.5×10^6^ ml^−1^ for Exp. 2, and 0.8×10^5^ ml^−1^ and 1.0×10^6^ ml^−1^ for Exp. 3, respectively. Overall, temperature significantly affected the temporal development of prokaryotic (Mann-Whitney *U* test: variation of *U* = 3.32–6.92, variation of *p* = <0.0001–0.0008) and viral abundance (Mann-Whitney *U* test: variation of *U* = 2.43–6.24, variation of *p* = <0.0001–0.0150). Thus, for all experiments, the deviation from initial prokaryotic and viral abundances was smaller at 4°C as compared to 21°C (Table 1, Figure 1). However, the effects of NaN_3_ on abundances of prokaryotes and viruses varied among the experiments (Tables 2–3).

**Table 1:** Deviation from initial prokaryotic and viral abundance.

Exp.	Treatment	Deviation in abundance				Days after start			
		Prokaryotes		Viruses		Prokaryotes		Viruses	
		Avg	SD	Avg	SD	±5%	±10%	±5%	±10%
1	21°C	120.3	122.3	63.4	41.1	1	1	1	1
	21°C, 0.005%	131.3	86.7	164.9	119.0	1	1	1	1
	21°C, 0.01%	215.3	120.7	483.3	646.6	1	1	1	1
	4°C	27.3	23.2	127.7	110.9	1	3	1	1
	4°C, 0.005%	6.7	4.8	124.8	48.7	4	6	1	1
	4°C, 0.01%	−1.2	1.4	25.7	21.5	>7	>7	1	1
2	21°C	96.0	196.1	0.1	58.5	1	1	1	1
	21°C, 0.005%	118.0	79.8	8.2	14.5	1	1	1	1
	21°C, 0.01%	147.3	48.4	77.4	56.1	1	1	1	1
	4°C	66.4	49.4	−8.2	17.9	1	3	2	2
	4°C, 0.005%	−2.9	8.4	−1.5	37.2	1	5	1	1
	4°C, 0.01%	−8.3	6.2	−25.5	11.7	2	2	1	1
3	21°C	288.5	317.3	72.2	34.5	1	1	1	1
	21°C, 0.005%	71.0	88.9	111.4	41.5	1	1	1	1
	21°C, 0.01%	−8.2	20.2	48.8	9.8	1	1	1	1
	4°C	−12.2	11.3	8.3	7.9	1	1	1	2
	4°C, 0.005%	−13.1	7.6	19.6	8.7	1	1	1	1
	4°C, 0.01%	−13.0	7.3	14.3	8.8	1	1	1	3

Average (Avg in % of initial abundance) and standard deviation (SD) of the changes in prokaryotic and viral abundance relative to initial abundances for all treatments (temperature, NaN_3_ concentration) during 7 days of incubation. The table also gives the number of days until prokaryotic and viral abundance changed more than ±5% and ±10% relative to initial abundances.

**Table 2:** Differences in the deviation from initial prokaryotic abundance among treatments.

	21°C, 0.005%			21°C, 0.01%			4°C			4°C, 0.005%			4°C, 0.01%		
	1	2	3	1	2	3	1	2	3	1	2	3	1	2	3
21°C	–	–	–	–	–	*	–	–	*	–	–	*	–	–	*
21°C, 0.005%				–	–	–	*	–	–	*	*	–	*	*	–
21°C, 0.01%							*	*	–	*	*	–	*	*	–
4°C										*	*	–	*	*	–
4°C, 0.005%													*	–	–

The table shows the results of multiple Mann-Whitney tests to test for significant differences in the deviation from initial prokaryotic abundance among the treatments. Statistically significant differences (Bonferroni-corrected: p≤0.0083) are indicated by ‘*’.

**Table 3:** Differences in the deviation from initial viral abundance among treatments.

	21°C, 0.005%			21°C, 0.01%			4°C			4°C, 0.005%			4°C, 0.01%		
	1	2	3	1	2	3	1	2	3	1	2	3	1	2	3
21°C	*	–	–	*	*	–	–	–	*	*	–	*	*	–	*
21°C, 0.005%				–	*	*	–	–	*	–	–	*	*	*	*
21°C, 0.01%							–	*	*	–	*	*	*	*	*
4°C										–	–	–	*	–	–
4°C, 0.005%													*	–	–

The table shows the results of multiple Mann-Whitney tests to test for significant differences in the deviation from initial viral abundance among the treatments. Statistically significant differences (Bonferroni-corrected: p≤0.0083) are indicated by ‘*’.

Consistent differences were found in Exps. 1–2, where the deviation from initial prokaryotic abundance was significantly smaller at 4°C compared to 21°C-0.01% NaN_3_ (Tables 1–2; Figure 1). Also, the deviation of prokaryotic abundance from initial conditions in Exps. 1–2 was significantly smaller at 4°C-0.005% NaN_3_ and 4°C-0.01% compared to 21°C-0.005%, 21°C-0.01%, and 4°C (Tables 1–2; Figure 1). However, in Exp. 3 the deviation from the original prokaryotic abundance was significantly higher only at 21°C compared to 21°C-0.01% NaN_3_, 4°C, 4°C-0.005%, and 4°C-0.01%, no further differences were found (Tables 1–2). Based on average values alone, a trend towards higher deviations from initial prokaryotic abundance from 21°C to 21°C-0.005% NaN_3_ and 21°C-0.01% in Exps. 1–2 was observable, however, this trend was reversed in Exp. 3 (Table 1). Changes in prokaryotic and viral abundance were positively correlated in Exp. 1 (*r* = 0.57, *p*<0.0001) and Exp. 2 (*r* = 0.67, *p*<0.0001), but only weakly in Exp. 3 (*r* = 0.20, *p* = 0.0369). Water for Exp. 3 was retrieved from 2000 m depth and initial prokaryotic abundance was an order of magnitude lower as compared to Exps. 1–2. Thus, differences in the prokaryotic communities between surface and deep samples might be responsible for the varying effects of NaN_3_.

The influence of NaN_3_ on the deviation from initial viral abundances was not consistent among experiments (Tables 1 and 3). In Exp. 1, the smallest change in viral abundance was found at 4°C-0.01% NaN_3_ (Tables 1 and 3; Figure 1). The data for Exps. 2–3 are not significantly different among all treatments, however, low deviations from initial viral abundances were detected at 21°C in Exp. 2, and at 4°C in Exp. 3 (Tables 1 and 3). In Exp. 2, the highest deviation from initial viral abundance was detected at 21°C-0.01% NaN_3_ (Tables 1 and 3). Differences between treatments were not consistently significant, however, high deviations from initial viral abundances were detected at 21°C-0.01% NaN_3_ in Exp. 1 (Figure 1), and at 21°C-0.005% NaN_3_ in Exp. 3 (Tables 1 and 3). Thus, the data suggest that the addition of NaN_3_ appears to stimulate virus production at 21°C.

### Conclusions

Occasionally, it took more than one day for deviations from initial viral abundance to exceed ±5% or ±10% in Exps. 2–3 (Table 1). However, the influence of NaN_3_ on the temporal development of viral abundance was variable among experiments. For surface samples (Exps. 1–2, Fig. 1), a final concentration of 0.01% (wt./vol.) NaN_3_ in combination with storage at 4°C resulted in stable numbers of prokaryotes for 2–7 days (Table 1). However, this was not reproducible in Exp. 3 (Table 1), conducted with water from 2000 m depth. In summary, we conclude that NaN_3_ is not a viable alternative to preserve prokaryotes and viruses in marine samples.

## Materials and Methods

### Ethics Statement

No specific permits were required for the described field studies. Sampling locations are not privately-owned or protected and sampling did not involve endangered or protected species.

### Sampling and experimental set-up

Water samples (1 L; Niskin bottles) were retrieved from 5 m and 2000 m depth at the DYFAMED time series station (43°25′N, 07°52′E), and surface water was retrieved with a sterile glass bottle from the Bay of Villefranche (France). Within one hour of sampling, the untreated water samples were dispensed into 50 mL polycarbonate tubes. For each sample (DYFAMED 5 m: Exp. 1; Bay of Villefranche: Exp. 2; DYFAMED 2000 m: Exp. 3), the following 6 treatments, each in triplicates, were set-up: 21°C, 21°C and 0.005% (weight:volume) NaN_3_, 21°C and 0.01% NaN_3_, 4°C, 4°C and 0.005% NaN_3_, and 4°C with 0.01% NaN_3_. The experiments were incubated in the dark for 7 days.

### Prokaryotic and viral abundance

Samples (1.8 mL) for prokaryotic and viral abundance were retrieved every 24 hours, fixed with glutaraldhyde (0.5% final concentration) in the dark for 15 min, flash-frozen in liquid nitrogen, and stored at −80°C until analysis. Upon thawing, the samples were stained with SYBR Green I (Invitrogen-Molecular Probes) and the abundance of prokaryotes and viruses was determined on a FACSCalibur (BD Biosciences) flow cytometer as previously described [3], [7].

### Statistical analyses

Kruskal-Wallis tests were used to test for significant differences among the treatments; significant results were further explored using Mann-Whitney pairwise comparisons. Spearman rank correlation coefficients (*r*) were used to test for significant correlations between the deviations from initial prokaryotic and viral abundance. Statistical tests were assumed significant at *p*-values≤0.05. In case of multiple comparison tests, *p*-values were corrected according to Bonferroni [16].
